# High NRF2 Levels Correlate with Poor Prognosis in Colorectal Cancer Patients and with Sensitivity to the Kinase Inhibitor AT9283 In Vitro

**DOI:** 10.3390/biom10101365

**Published:** 2020-09-25

**Authors:** Laura Torrente, Gunjit Maan, Asma Oumkaltoum Rezig, Jean Quinn, Angus Jackson, Andrea Grilli, Laura Casares, Ying Zhang, Evgeny Kulesskiy, Jani Saarela, Silvio Bicciato, Joanne Edwards, Albena T. Dinkova-Kostova, Laureano de la Vega

**Affiliations:** 1 Jacqui Wood Cancer Centre, Division of Cellular Medicine, School of Medicine, University of Dundee, Dundee DD1 9SY, UK; laura.torrentefernandez@moffitt.org (L.T.); g.maan@dundee.ac.uk (G.M.); a.w.y.jackson@dundee.ac.uk (A.J.); l.casaresperez@dundee.ac.uk (L.C.); Y.F.Zhang@dundee.ac.uk (Y.Z.); A.DinkovaKostova@dundee.ac.uk (A.T.D.-K.); 2 Department of Cancer Physiology, H. Lee Moffitt Cancer Center and Research Institute, Tampa, FL 33612, USA; 3 Unit of Gastrointestinal Oncology and Molecular Pathology, Institute of Cancer Sciences, College of Medical, Veterinary, and Life Sciences, University of Glasgow, Glasgow G61 1QH, UK; 2421235R@student.gla.ac.uk (A.O.R.); Jean.Quinn@glasgow.ac.uk (J.Q.); Joanne.Edwards@glasgow.ac.uk (J.E.); 4 Department of Life Sciences, University of Modena and Reggio Emilia; via G, Campi 287, 41125 Modena, Italy; andrea.grilli2@unimore.it (A.G.); silvio.bicciato@unimore.it (S.B.); 5 Institute for Molecular Medicine Finland (FIMM), University of Helsinki, Tukholmankatu 8, FI-00290 Helsinki, Finland; evgeny.kulesskiy@helsinki.fi (E.K.); jani.saarela@helsinki.fi (J.S.); 6 Departments of Medicine and Pharmacology and Molecular Sciences, Johns Hopkins University School of Medicine, Baltimore, MD 21205, USA

**Keywords:** NRF2, colorectal cancer, drug screening

## Abstract

Aberrant hyperactivation of nuclear factor erythroid 2 (NF-E2) p45-related factor 2 (NRF2) is a common event in many tumour types and associates with resistance to therapy and poor patient prognosis; however, its relevance in colorectal tumours is not well-established. Measuring the expression of surrogate genes for NRF2 activity in silico, in combination with validation in patients’ samples, we show that the NRF2 pathway is upregulated in colorectal tumours and that high levels of nuclear NRF2 correlate with a poor patient prognosis. These results highlight the need to overcome the protection provided by NRF2 and present an opportunity to selectively kill cancer cells with hyperactive NRF2. Exploiting the CRISPR/Cas9 technology, we generated colorectal cancer cell lines with hyperactive NRF2 and used them to perform a drug screen. We identified AT9283, an Aurora kinase inhibitor, for its selectivity towards killing cancer cells with hyperactive NRF2 as a consequence to either genetic or pharmacological activation. Our results show that hyperactivation of NRF2 in colorectal cancer cells might present a vulnerability that could potentially be therapeutically exploited by using the Aurora kinase inhibitor AT9283.

## 1. Introduction

The transcription factor NRF2 (nuclear factor erythroid 2 (NF-E2) p45-related factor 2, encoded by *NFE2L2*) is the master regulator of oxidative stress responses, which allows adaptation and survival during stress conditions. In normal cells under nonstress conditions, NRF2 levels and activity are kept low by its fast proteasomal degradation, which is principally facilitated by KEAP1 (a substrate adaptor for a Cul3-based E3 ubiquitin ligase) [1,2]. Although NRF2 is a well-characterised cytoprotective factor in normal cells, its sustained activation protects tumour cells against chemo- and radiation therapies and promotes metabolic switches that support cell proliferation and tumour growth [3,4,5,6,7]. Sustained activation of the NRF2 pathway is mainly due to either loss-of-function (LOF) mutations in *KEAP1* [3,8] or gain-of-function (GOF) mutations in *NFE2L2* [8,9]. This has been shown in a variety of tumours [3,8]. Somatic mutations of *NRF2/KEAP1* are particularly prevalent in lung adenocarcinoma (LuAD) and squamous cell carcinoma (LuSCC) [8,10,11,12]. It is estimated that 34% LuSCC and 18% of LuAD patients harbour mutations in *NRF2* or *KEAP1*, which correlates with poor survival [11,13]. Additionally, aberrant activation of NRF2 in cancer cells also occurs through alternative mechanisms, including the oncogene-induced transcription of *NRF2* via KRAS, BRAF, MYC activation [14,15] or epigenetic silencing of *KEAP1* [16,17].

Altogether, this shows that the aberrant activation of the NRF2 pathway might be a common pro-oncogenic event in many cancer types, and thus, the identification of ways to overcome the protection provided by NRF2 is a desirable goal. Despite efforts to identify selective NRF2 inhibitors, chemical inhibitors of NRF2 identified to date lack potency and/or specificity, and none have entered clinical trials [18,19,20,21,22,23]. Furthermore, the safety of a systemic treatment with an NRF2 inhibitor for patients is uncertain due to the critical cytoprotective role of NRF2 in normal cells and its important role in the immune response [24,25,26]. An alternative way to overcome the NRF2-mediated aggressiveness in cancer is to identify vulnerabilities associated with NRF2 hyperactivation. Since such an approach is not expected to affect normal cells, it will be more specific and, consequently, safer than systemic NRF2 inhibition.

In colorectal cancer, genetic alterations of *KEAP1* or *NFE2L2* are rare (less than 2%). The activation of NRF2 in colorectal cancer has been shown to happen through at least two different mechanisms: hypermethylation of the *KEAP1* promoter and deubiquitination of NRF2 [16,27]. However, the actual contribution of NRF2 to tumour development and therapeutic responses in colorectal cancer remains unclear, with conflicting data regarding NRF2 levels, the relevance of its localisation and correlation with prognosis [28,29,30,31,32,33,34]. Further, the use of potentially unspecific NRF2 antibodies [35] and the lack of antibody validation for in immunohistochemistry analyses call the results of some studies into question. This is in contrast with lung cancer, where, in addition to the existence of well-characterised cell lines harbouring mutant hyperactive NRF2 (e.g., the lung cancer cell line A549), several models have been developed to study NRF2 hyperactivation, and a number of associated vulnerabilities have been identified by using KEAP1-deficient or loss-of-function mutant models [36,37,38,39]. In addition to NRF2, KEAP1 also interacts with other proteins [40], but the number of studies addressing the role of NRF2 hyperactivation in the presence of wild-type KEAP1 is limited.

To fill these gaps, in this study, we first investigated the activation of the NRF2 pathway and its correlation with prognosis in colorectal tumours by performing a comprehensive in silico analysis. The activation of NRF2 in colorectal tumours compared to matched normal tissue was then validated in samples from patients. Furthermore, we generated isogenic colorectal DLD1 cell lines harbouring gain-of-function (GOF) mutations in *NRF2*. Using this model, we screened a panel of 528 drugs for their ability to kill DLD1 cells with hyperactive NRF2 whilst sparing DLD1 cells expressing “normal” levels of NRF2. Remarkably, we found that DLD1 NRF2-GOF cells are more sensitive to a number of Aurora kinase inhibitors compared to DLD1 control cells. Furthermore, we validated the best hit from the drug screening, the Aurora kinase inhibitor AT9283, and showed that the genetic and pharmacological activation of NRF2 increases the drug sensitivity to AT9283.

## 2. Materials and Methods

### 2.1. Cell Culture

All DLD1 cell lines were grown in Dulbecco’s modified Eagle’s medium (DMEM) containing 10% Fetal Bovine Serum (FBS) at 37 °C and 5% CO_2_. The cell lines were validated by Short Tandem Repeat (STR) profiling and were routinely tested to ensure that they were mycoplasma-negative. CRISPR-edited NRF2 knockout (NRF2-KO) and NRF2 gain-of-function (NRF2-GOF) DLD1 cell lines were produced as previously described [41].

### 2.2. Antibodies and Reagents

Antibody recognising NRF2 (D1Z9C) for Western blotting analyses was obtained from Cell Signaling Technology (Danvers, MA, USA) and for immunohistochemistry (IHC) from Santa Cruz Biotechnology (sc-365949) (Dallas, TX, USA). Anti-beta-actin antibody (C4) was from Santa Cruz Biotechnology. Horseradish peroxidase (HRP)-conjugated secondary antibodies were from Life Technologies (Carlsbad, CA, USA). AT9283 was purchased from ApexBio Technology (Houston, TX, USA). Dimethyl sulfoxide (DMSO) was from Sigma-Aldrich (Dorset, UK). R,S-sulforaphane (SFN) was purchased from LKT Laboratories (St. Paul, MN, USA). The (±)-TBE-31 and HB229 were kindly provided by Honda and Wells, respectively [42,43].

### 2.3. Quantitative Real-Time PCR (rt-qPCR)

The use of human tissues was approved by the Tayside Tissue Bank Research Ethics Committee, a devolved subcommittee of the Tayside Committee on Medical Research Ethics.

RNA from tissue or cells was extracted using a RNeasy kit (QIAGEN, Hilden, Germany). RNA was reverse-transcribed to cDNA using an Omniscript RT kit from QIAGEN (Hilden, Germany) supplemented with a RNase inhibitor, according to the manufacturer’s instructions. The resulting cDNA was analysed using TaqMan Universal Master Mix II (Life Technologies, Carlsbad, CA, USA). Gene expression was determined using an Applied Biosystems 7300 real-time PCR system by the comparative ΔΔCT method. All experiments were performed at least in triplicates, and data were normalised to the housekeeping gene HPRT1 (in cells) or β-actin (in tissue). The primers used were obtained from Thermo Fisher Scientific (Waltham, MA, USA) as follows: NQO1 (Hs00168547_m1), AKR1B10 (Hs00252524_m1), NFE2L2 (Hs00975961_g1), HPRT1 (Hs02800695_m1) and β-actin (Hs01060665_g1).

### 2.4. Focused Oxidative Stress Pathway Expression Analysis

The Human Oxidative Stress RT2 Profiler PCR Array (QIAGEN) profiled the expression of 84 genes related to oxidative stress (ID 330231). The list of genes can be obtained from the QIAGEN webpage. The analysis was performed in triplicates, including several internal controls and housekeeping genes, and analysed using the QIAGEN in-house software (Software Version 2.0.4).

### 2.5. Cell Lysis Protocol and Western Blotting

Cells were washed and harvested in ice-cold phosphate-buffered saline (PBS), lysed in Radioimmunoprecipitation assay buffer (RIPA) buffer and sonicated. Lysates were cleared by centrifugation for 15 min at 4 °C. The supernatant was mixed with sodium dodecyl sulfate (SDS) sample buffer and boiled for 5 min. Equal amounts of protein were separated by SDS-PAGE (polyacrylamide gel electrophoresis), followed by semi-dry blotting to a polyvinylidene difluoride membrane (PVDF, Thermo Fisher Scientific). After blocking of the membrane with 5% (*w*/*v*) Tris buffered saline with Tween 20 (TBST) nonfat dry milk, primary antibodies were added. Appropriate secondary antibodies coupled to horseradish peroxidase were detected by enhanced chemiluminescence using Clarity™ Western ECL (enhanced chemiluminescent) Blotting Substrate (Bio-Rad, Hercules, CA, USA).

### 2.6. Drug Screening

Drug sensitivity and resistance testing (DSRT) [44] was performed on DLD1 NRF2 wild-type (WT) and GOF cell lines in collaboration with The FIMM High-Throughput Biomedicine Unit at the University of Helsinki (Helsinki, Finland). The compound library included 528 substances consisting of conventional chemotherapeutics and a broad range of targeted oncology compounds. The compounds were dissolved in dimethyl sulfoxide or water and dispensed on 384-well plates (Corning, Corning, NY, USA). Each compound was plated at 5 concentrations covering a 10,000-fold concentration range. The cells were incubated with the compounds, and after 72 h, cell toxicity and viability were measured with CellToxGreen and CellTiter-Glo assays, respectively (Promega, Fitchburg, WI, USA) using a Pherastar FS (BMG Labtech, Offenburg, Germany) plate reader. The data were normalised to negative control wells (dimethyl sulfoxide only) and positive control wells containing 100-μM benzethonium chloride, which effectively kills all cells using a Breeze analysis pipeline [45]. To assess quantitative drug profiles for each sample, we calculated a drug sensitivity score (DSS) based on the measured dose-response curves [46]. DSS is an integrative and robust drug response metric based on the normalised area under the curve, which considers all four curve-fitting parameters in the logistic model. The complete screening data is available in Supplementary Tables S1–S4.

### 2.7. Cell Viability Assays

Cells were seeded at a density of 2000 cells/well in 96-well plates, using three to five replicates for each treatment condition. On the next day, cells were treated with either 0.1% DMSO or different concentrations of AT9283 ranging from 10 nM to 10,000 nM. Treated cells were incubated for five days at 37 °C.

Alamar Blue: Cell viability was determined by adding 20 µL of Alamar blue dye (Thermo Fischer Scientific) to 200 µL of cell growth medium (DMEM). The plates were incubated with the dye for 3–6 h at 37 °C, and the fluorescence (excitation at 570 nm and emission at 585 nm) was determined using a standard plate reader.

Crystal Violet: Media was removed from plate wells and cells washed with PBS. Cells were fixed using cold 4% paraformaldehyde (PFA) and kept on ice for twenty minutes. PFA was discarded, and after washing with PBS, cells were incubated with 0.1% crystal violet at room temperature. Twenty minutes later, crystal violet was removed, and the wells rinsed multiple times with dH_2_O, until no blue dye could be seen in the rinse. Plates were then left to dry at room temperature before adding 10% acetic acid. Absorbance was read at 590–600 nm.

### 2.8. In Silico Quantification of the NRF2 Activity in Colon and Rectal Tumours

To quantify in silico the NRF2 activity, we used the gene expression profiles of colon carcinoma and rectal carcinoma tissues and of their normal counterparts obtained from the TCGA (The Cancer Genome Atlas Program) project as harmonised datasets. Specifically, the first dataset was composed of 478 primary tumours and 41 normal tissues, whereas the latter of 166 primary tumours and 10 normal tissues. Their gene expression data were downloaded as fragments per kilobase per million mapped reads (FPKM) normalised expressions using the TCGAbiolinks package [47] (version 2.15.3) in R 3.5.0 (https://cran.r-project.org/). When present, multiple Ensembl IDs matching the same gene symbol were collapsed to their median gene expression. The differential activity of NRF2 in tumour and normal tissues was tested using the NRF2 target gene NQO1 and the combined use of five well-known NRF2 validated target genes (“5-gene signature”). Additionally we also tested (a) a gene set formed by 32 direct NRF2 targets (“NRF2 multi-tumour” [48]) identified in bladder urothelial carcinoma, lung squamous cell carcinoma, head-neck squamous cell carcinoma and uterine corpus endometrial carcinoma and (b) a signature formed by 20 NRF2 target genes identified in lung cancer cells (“NRF2 lung” [49]). Signature scores for each gene set were obtained summarising the standardized expression levels of the signature genes into a combined score with a zero mean [50]. Differences between signature scores in tumour and normal samples were calculated using a Welch’s *t*-test in R.

### 2.9. Immunohistochemistry

Immunohistochemistry was performed to assess the protein levels of NRF2 using the anti-NRF2 antibody from Santa Cruz (sc-365949) on a previously constructed tissue microarray (TMA) consisting of 356 patients who underwent elective, potentially curative, resection for stage I-IV colorectal cancer between 1997 and 2007 from Glasgow Royal Infirmary, Western Infirmary Glasgow and Stobhill Hospital (Glasgow, UK). The selection criteria excluded patients who had neoadjuvant therapy, died within 30 days of surgery or had metastasis. Local ethical approval was obtained from the West of Scotland Research Ethics Committee (16/ws/0207; 14/03/2017), and tissue for this analysis was obtained from the National Health Service Greater Glasgow and Clyde Tissue Biorepository (number 416; 10/10/2017) (Glasgow, UK).

Antibody specificity was confirmed using cell pellets prepared from WT and NRF2-GOF DLD1 cells. TMA sections (2.5 µm) were dewaxed by immersion in Histoclear, then rehydrated using a series of alcohols. Heat-induced antigen retrieval was performed in a solution of Tris-EDTA (ethylenediaminetetraacetic acid) pH9; after which, the sections were incubated in 3% hydrogen peroxide. Nonspecific binding was blocked by incubation in 1.5% normal horse serum before being incubate with NRF2 antibody (1:100) overnight at 4 °C. Staining was visualised with ImmPress and 3,3′-diaminobenzidine (DAB) (both Vector Laboratories, Peterborough PE26XS, UK). Tissue was counterstained using Harris haematoxylin before being dehydrated and mounted using DPX (dibutyl phthalate in xylene).

The stained TMA sections were scanned using Hamamatsu NanoZoomer (Welwyn Garden City, Hertfordshire, UK) at 20x magnification. Visualisation was carried out using Slidepath Digital Image Hub version 4.0.1 (Slidepath, Leica Biosystems, Milton Keynes, UK). The weighted histoscore method was employed to assess the protein expression by observer 1 (AR), with each protein being scored by a second independent observer (JE) who scored 10% of all tumours to ensure consistency across the study. Interclass correlation coefficients of greater than 0.8 were deemed acceptable and Bland Altman plots carried out to ensure no scoring bias. All observers were blinded to the clinical and pathological characteristics. Histoscores were calculated using the following formula: Total score = (% of unstainted tumor cells × 0) + (% of weakly stained tumor cells × 1) + (% of moderately stained tumor cells × 2) + (% of strongly stained tumor cells ×3), giving a range of 0 (minimum) to 300 (maximum). The results were considered discordant if the histoscores differed by more than 50. Cytoplasmic and nuclear expressions were calculated separately.

## 3. Results and Discussion

### 3.1. Analysis of the Status of the NRF2 Pathway in Colorectal Tumours

**Figure 1:**
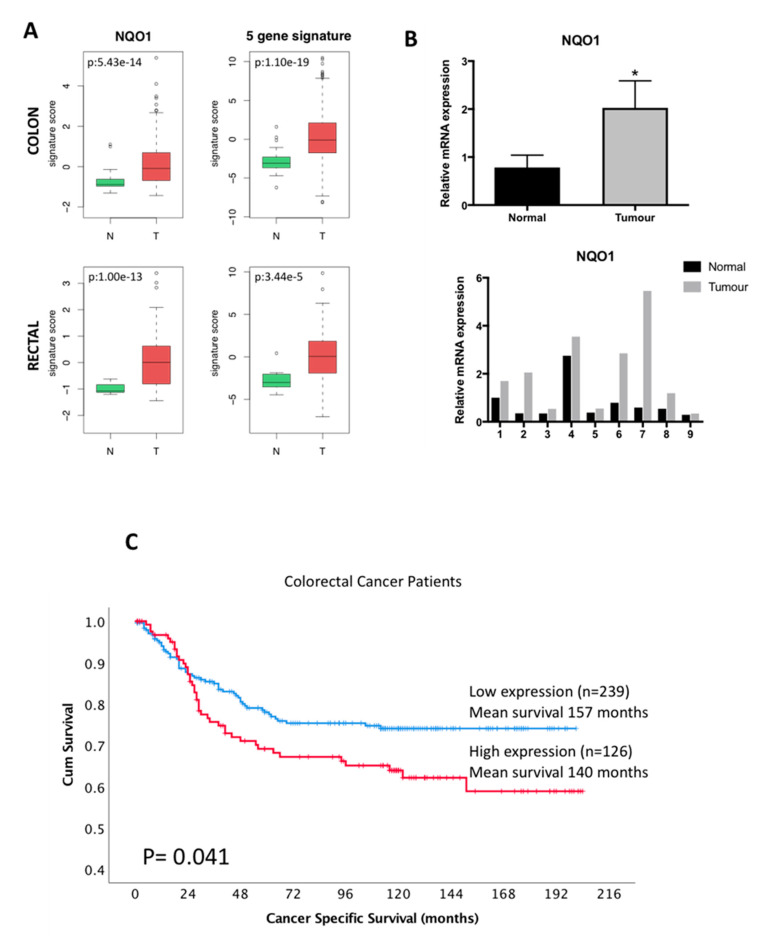
Relevance of the nuclear factor erythroid 2 (NF-E2) p45-related factor 2 (NRF2) pathway in colorectal tumours in vivo. (**A**) Bioinformatic analysis of the expression of various NRF2 target genes in normal and tumour tissues. The activity of NRF2 in colon (upper row) and rectal (lower row) cancers (T) and normal (N) tissues of the TCGA project was assessed through the expression of the NRF2 target genes *NQO1* (left) and through the combined score from the expression of *NQO1, GPX2, TXNRD1, GCLC* and *GCLM* (right). p: *p*-value of the Welch’s *t*-test. (**B**) *NQO1* expression in matched normal and tumour colorectal tissue quantified using real-time PCR. Figures show the combined data (upper panel) or individual patients (lower panel). The data were normalised using β-actin as an internal control. Data represent means ± SD (*n* = 9). The differences between the tumour and the normal tissue for NQO1 were statistically significant (paired *t*-test analysis) (* *p* = 0.0401). (**C**) Kaplan-Meier plots showing differential cancer-specific survival for colorectal patients with high or low nuclear NRF2 levels (*p* = 0.041).

To investigate the status of NRF2 activity in colorectal tumours, we first interrogated large databases of publicly available gene expression data from the TCGA project. Considering that the NRF2 pathway is mainly activated by NRF2 stabilisation, *NRF2* mRNA levels do not capture the status of NRF2 activity. Therefore, and as there are no NRF2 transcriptional signatures validated in colorectal tumours, we used well-known NRF2 target genes as surrogates for NRF2 activity: the prototypical NRF2 target gene *NQO1*, which is often used as a surrogate for NRF2 activity, and, also, a combination of five well-characterised and validated NRF2 target genes: *NQO1, GPX2, TXNRD1, GCLC* and *GCLM* (all these genes were upregulated in our in vitro model of colorectal NRF2-GOF cells). We found that both NQO1 and the combined NRF2 target genes were significantly enriched in colon tumours and rectal tumours when compared with normal tissue (Figure 1A), suggesting that the NRF2 pathway is activated in colorectal tumours. Additionally, we also tested two validated NRF2 signatures identified in other tumour types (Supplementary Figure S1A): (a) a NRF2 signature identified in bladder urothelial carcinoma, lung squamous cell carcinoma, head-neck squamous cell carcinoma and uterine corpus endometrial carcinoma [48] and (b) a NRF2 signature identified in lung cancer cells [49]. While the NRF2 lung cancer signature was not enriched in either dataset, the NRF2 multi-tumour signature was significantly upregulated in colon but not in rectal. Our data highlight the importance of the identification and validation of a NRF2 signature specific for colorectal tumours, as signatures from other tumour types might not be relevant.

To validate these in silico data, but restricted by the amount of material available, we compared the *NQO1* mRNA levels (as a surrogate for NRF2 activity) of nine chemo-naïve colorectal tumours and matched normal tissue samples (Figure 1B). Eight out of the nine sample pairs displayed significantly higher levels of *NQO1* in the tumours compared to their corresponding matched normal tissue, supporting our in silico data.

**Figure 2:**
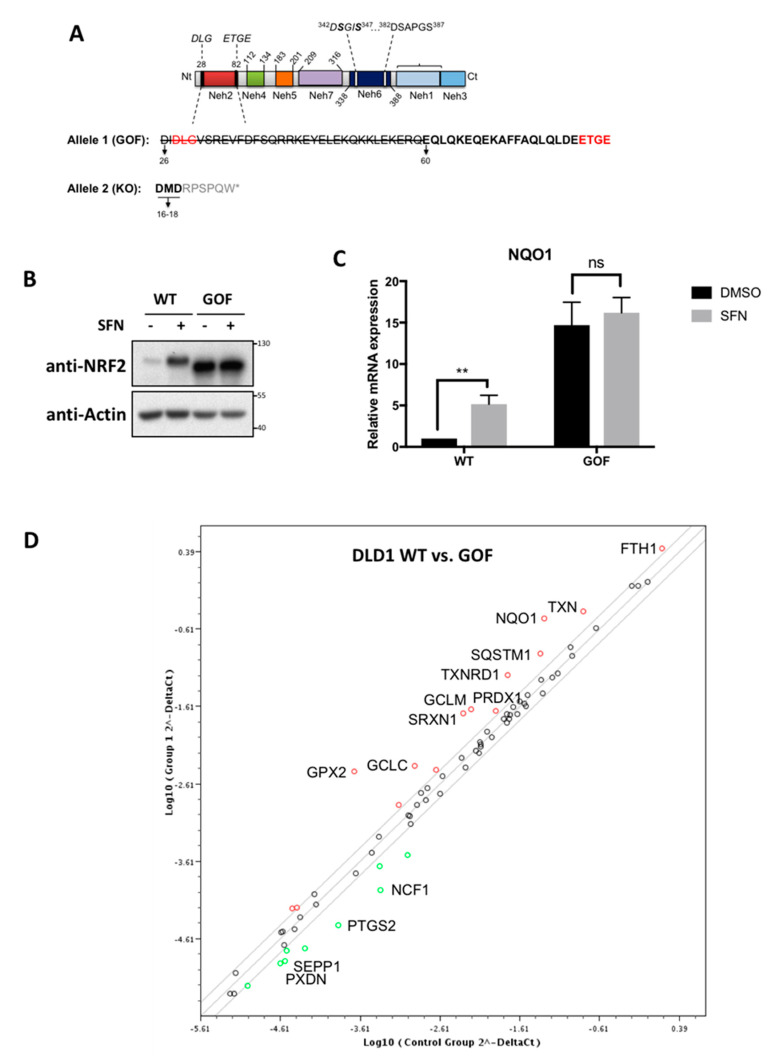
Validation of a new colorectal cancer NRF2-GOF (gain-of-function) in vitro model. (**A**) Schematic representation of the NRF2 domains and the sequencing on the NRF2-GOF clones. (**B**) Isogenic NRF2-WT (wild-type) and NRF2-GOF DLD1 cell lines were treated with either DMSO (−) or with 5 µM of sulforaphane (SFN) (+) for 3 h, and the protein levels of NRF2 were compared. (**C**) The mRNA levels for NQO1 in the indicated cell lines treated with dimethyl sulfoxide (DMSO) or with 5 µM of SFN for 16 h were quantified using real-time PCR. The data were normalised using β-actin as an internal control. Data represent means ± SD (*n* = 3) and are expressed relative to the WT DMSO cells. ** *p* ≤ 0.01. (**D**) Representation of the differential expression of oxidative stress-related genes in NRF2 WT versus NRF2-GOF. Highlighted either in red (upregulated) or in green (downregulated) are genes with more than 2-fold changes; only those with *p*-values < 0.05 were labelled (*n* = 3).

The next important question to answer was whether, similar to other cancer types, NRF2 hyperactivation correlates with a poor prognosis in colorectal cancer. To directly address this question, we analysed the protein levels of NRF2 on a tissue microarray (TMA) containing samples from 365 colorectal cancer patients and their association with prognosis. We tested the specificity of two different antibodies against human NRF2 in formalin-fixed paraffin-embedded (FFPE) cell pellets of NRF2-GOF and NRF2-KO DLD1 cells (see cell validation for NRF2-GOF DLD1 cells in Figure 2 and for NRF2-KO DLD1 cells [41]). First, we compared the intensity of the IHC staining of NRF2-KO versus NRF2-GOF cells using the NRF2 antibody 62,352 from Abcam (Cambridge, UK), which has been previously used in human colorectal tissue [51,52]. However, there was no differential intensity between the NRF2-KO and NRF2-GOF DLD1 cells, suggesting that the antibody was not specific to NRF2 (Supplementary Figure S1B, upper panels). We then validated the specificity of a second NRF2 antibody (sc-365949, Santa Cruz), which showed an intense nuclear signal in NRF2-GOF and no staining of NRF2-KO cells (Supplementary Figure S1B, lower panels). Using the data obtained from Santa Cruz antibody IHC staining (antibody validation shown in Supplementary Figure S1B,C), we analysed whether nuclear NRF2 levels (and, thus, the transcriptionally active population) impact the survival of colorectal cancer patients. Indeed, high protein levels of nuclear NRF2, correlated with a decreased survival of colorectal cancer patients (*p* = 0.041) (Figure 1C), with a mean difference in cancer-specific survival of 17 months between those patients with high NRF2 expressions versus those with low NRF2 expressions. In addition, nuclear NRF2 levels significantly correlated with both the proliferation index and defective DNA mismatch repair (MMR) status but not with other studied clinical factors (Supplementary Figure S1C).

Our data suggest that the NRF2 pathway is in fact activated in colorectal tumours, leading to poorer patient prognosis.

### 3.2. Generation and Validation of a New Colorectal Cancer NRF2-GOF Cell-Based Model

To generate a colorectal cancer cell-based model that allows us to study the effects of the constitutive activation of NRF2, we used the CRISPR/Cas9 system to generate isogenic stable cell lines. We designed a single guide RNA (sgRNA) that targets the DLG motif of endogenous NRF2. This motif is one of the two KEAP1-binding motifs within NRF2, and thus, its deletion disrupts the functional interaction between NRF2 and KEAP1. Cas9-mediated DNA cleavage may result in out-of-frame DNA repair, leading to NRF2-KO clones. However, most of the cell clones in which the DNA is repaired in-frame will result in NRF2-GOF clones (as described in [41]) due to a mutation or deletion of the KEAP1-binding sequence. These gain-of-function (GOF) deletions would functionally resemble some of the NRF2 mutations found in tumours [5,9,13].

To characterise the genetic alterations introduced by CRISPR into the DLD1 NRF2 GOF cell lines, we sequenced a single cell-derived clone (Figure 2A). The sequencing showed that one of the *NFE2L2* alleles was repaired in-frame, but the DLG motif was deleted, while the ETGE motif remained intact. The second allele was null for NRF2 expression, as it was not repaired in-frame, which led to an early stop codon. The resulting phenotype showed an activation of the NRF2 pathway, as demonstrated by the increased protein levels of NRF2 (Figure 2B) and the correspondingly higher mRNA levels of the target gene *NQO1* (Figure 2C); notably, these levels were much higher than those induced in response to the pharmacological NRF2 activator sulforaphane (SFN). A pathway-focused gene expression analysis for 86 antioxidant genes in NRF2-GOF versus NRF2-WT DLD1 cell lines (Figure 2D) further confirmed the upregulation of well-established NRF2 target genes. Using the same gRNA, we generated an isogenic NRF2-KO cell line and compared the mRNA levels of *NQO1* and *AKR1B10* of NRF2-WT, NRF2-KO and NRF2-GOF cells (Supplementary Figure S2). The mRNA data suggests that the gRNA used in this study is selective for the *NFE2L2* gene locus; hence, the phenotypes observed are not due to off-target effects.

Altogether, using the CRISPR/Cas9 system, we generated stable DLD1 cells harbouring the constitutive activation of NRF2 by truncating one of the domains required for KEAP1 binding. This cell line might be used as a tool to characterise the consequences of NRF2 activation in colorectal cancer.

### 3.3. Drug Screening

To identify drugs with selective cytotoxicity against colorectal cancer cells harbouring hyperactive NRF2, we used our newly generated cell-based model. We tested the cytotoxicity potential of 528 drugs (158 approved drugs, 285 investigational and 85 probes) in DLD1 cells with hyperactive NRF2 (NRF2-GOF) over DLD1 control cells (NRF2-WT). Two different analyses were performed to evaluate the cytotoxic potential of these drugs, including a cell viability assay (namely CellTiter-Glo, CTG) and cytotoxicity assay (CellTox-Green, CTX).

**Figure 3:**
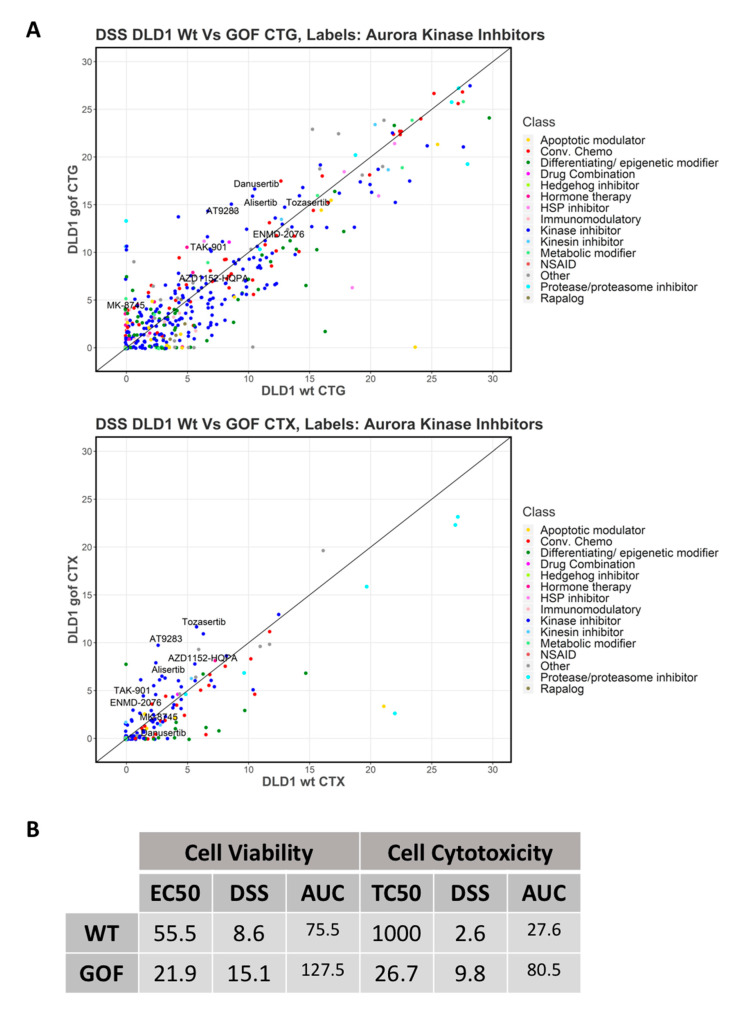
Synthetic lethality drug screening. (**A**) Scatter plots showing the drug sensitivity score (DSS) values of the Aurora kinase inhibitor compounds in the screening panel in DLD1 WT and GOF cells for (upper panel) cell viability (CellTiter-Glo, CTG) and for (lower panel) cell toxicity (CellTox Green, CTX) readouts. Labels show only Aurora kinase inhibitors. (**B**) Key drug response parameters of AT9283 in both WT and NRF2-GOF DLD1 cells are shown. TC_50_ (half-maximal toxic concentration), EC_50_ (half-maximal effective concentration), AUC (area under the curve) and DSS (drug sensitivity score).

From this screen, the drug sensitivity score (DSS), which is an integrative and robust drug response metric based on the normalised area under the curve (see Materials and Methods), was obtained. Only compounds that had a difference in DSS between NRF2-GOF and NRF2-WT higher that five in both analyses were considered positive hits. This analysis identified the Aurora kinase inhibitor AT9283 as the top candidate with selectivity against NRF2-GOF DLD1 cells (Figure 3A,B and Supplementary Figure S3A,B). AT9283 is a synthetic small heterocyclic molecule that potently inhibits several kinases, including Aurora A (3 nM), Aurora B (3 nM), JAK2 (1.2 nM), JAK3 (1.1 nM) and Abl (4.0 nM, T315I) [53]. Importantly, Alisertib, a well-characterised Aurora A kinase inhibitor, also displayed an increased selectivity against NRF2-GOF compared to the DLD1 control cells in both analyses performed (Figure 3A). Other Aurora kinase inhibitors tested, including Tozasertib and Danusertib, were significantly more potent against NRF2-GOF DLD1 cells in one of the analysis performed. The drug screen also included other known Aurora kinase inhibitors (TAK 901, ENMD 2076, AZD A552-HQPA and MK-8745) that, although they did not pass the set threshold, displayed a selective trend against NRF2-GOF cells. Altogether, these analyses suggest that AT9283 selectively kill DLD1 cells harbouring NRF2 hyperactivation through the inhibition of Aurora kinases.

AT9283 showed antimyeloma, antilymphoma, antileukaemia and anticolorectal cancer activity in preclinical studies [54,55,56,57,58,59], and its safety and efficacy against myeloma, lymphoma and leukaemia has been tested in various Phase I and II clinical trials [60,61,62,63,64,65,66]. Therefore, we focused our subsequent studies on AT9283.

### 3.4. Validation of the Selectivity of AT9283 against Active NRF2

**Figure 4:**
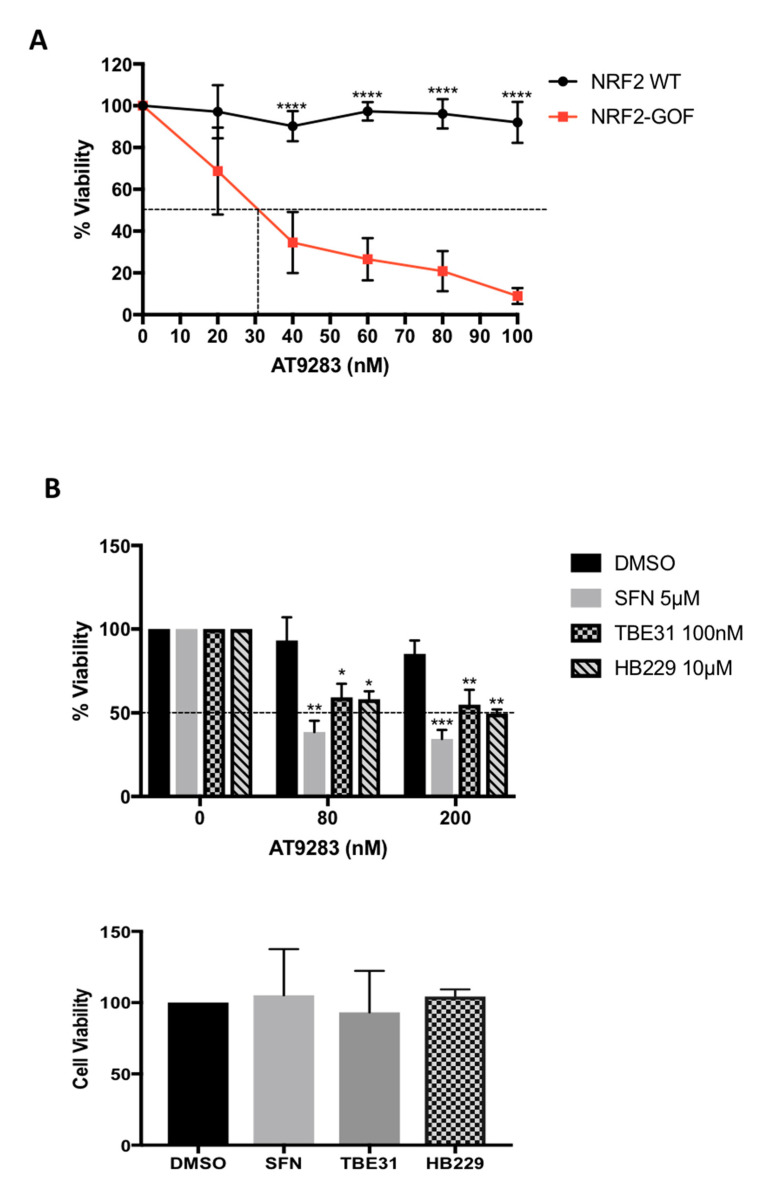
Validation of the selectivity of AT9283 against active NRF2. (**A**) NRF2-WT and GOF DLD1 cells were exposed to increasing concentrations of AT9283, as indicated. After three days, cell viability was measured using Alamar blue. Data represent means ± SD (*n* = 4) and are expressed relative to the DMSO control, which was set as 100%. (**B**) Upper panel: DLD1 cells were pretreated with the indicated concentrations of NRF2 inducers or with DMSO, as indicated. After 24 h, fresh media with either DMSO or 80-nM or 200-nM AT9283 was added, and NRF2 inducers were replenished. On the next day, the NRF2 inducers were added again, and 24 h later, the cell viability was measured using Alamar blue (*n* = 3). The viability of all control cells (without AT9283) were set as 100%. Lower panel: The cell viability of control cells (without AT9283) treated with the different NRF2 inducers is shown. * *p* ≤ 0.05, ** *p* ≤ 0.01, *** *p* ≤ 0.001, **** *p* ≤ 0.0001.

To validate the top hit from the screen, we tested the effect of AT9283 on the viability of NRF2-WT and NRF2-GOF DLD1 cells after three days, using the Alamar blue assay as the readout (Figure 4A). This assay confirmed the increased (by ~10-fold) sensitivity of the NRF2-GOF compared to NRF2-WT cells: AT9283 had a half maximal inhibitory concentration (IC_50_) of 28 nM in NRF2-GOF cells versus an IC_50_ of 320 nM in NRF2-WT cells. We further confirmed the effects of AT9283 on cell survival using crystal violet staining, an ATP (adenosine triphosphate)-independent assay (Supplementary Figure S4A), and, also, CellTiter-Glo, a luminescent ATP-dependent assay (Supplementary Figure S4B). In both cases, the cells were incubated for five days with the drugs, and thus, the reduced sensitivity was observed.

This analysis showed that AT9283 successfully “discriminates” between cell lines based on their NRF2 activity, with cells with high levels of NRF2 activity (GOF) having an increased sensitivity to AT9283.

To evaluate the potential therapeutic value of this selectivity against cells with high levels of NRF2, and to further ensure that the observed effect is dependent on NRF2 activation and is not a consequence of clonal artefacts, we tested whether the selective cytotoxicity could be recapitulated by using pharmacological NRF2 activation. For this, we compared the effect on the viability of NRF2-WT cells of three NRF2 activators that differ in potency and mechanism of action. Sulforaphane (SFN) and TBE31 are two well-characterised electrophilic compounds that react with cysteine sensors (primarily C151) of KEAP1, impairing its ability to target NRF2 for degradation [66]. By contrast, HB229 is a nonelectrophilic small molecule that disrupts the KEAP1–NRF2 protein complex [43]. When NRF2-WT cells were treated with the NRF2 activators, we observed a significant sensitisation towards the toxicity of AT9283 (Figure 4B, upper panel). Critically, the effect on cell viability was completely dependent on AT9283 acting together with the NRF2 activator, as, at these concentrations, neither AT9283 (Figure 4A,B) nor any of the NRF2 activators by themselves (Figure 4B, lower panel) affected the viability of the cells. Of note, the sensitisation obtained with the pharmacological activation of NRF2 was not as dramatic as the one observed with genetic activation. This is consistent with the fact that the magnitude of the pharmacological activation of the pathway (measured by *NQO1* levels) is significantly lower than that obtained with genetic modulation (Figure 2C and Supplementary Figure S4C). Nevertheless, these results demonstrate that, similar to the genetic, the pharmacological activation of NRF2 also sensitises DLD1 cells towards AT9283-mediated killing and further suggests that AT9283 could be used in combination with an NRF2 activator to sensitise cells with normal NRF2 levels.

## 4. Conclusions

In conclusion, we showed that, similar to other cancer types, NRF2 is hyperactivated in colorectal tumours, which correlates with a poor prognosis. Furthermore, using a new model of a colorectal cancer cell line with hyperactive NRF2, we identified a family of compounds (Aurora kinases inhibitors) and, especially, the kinase inhibitor AT9283 for its selectivity against colorectal cancer cells with hyperactive NRF2. Our study presents proof of concept data that the use of in vitro models of hyperactive NRF2 can identify drugs with specificity against cells with high NRF2 levels. This could have clinical relevance for the treatment of tumours with hyperactive NRF2, as well as tumours with normal NRF2, when combined with a pharmacological NRF2 activator. Further characterisation of this preferential selectivity in other tumour types and in in vivo models is necessary to confirm the clinical potential of this approach.

## Supplementary Materials

The following are available online at https://www.mdpi.com/2218-273X/10/10/1365/s1: Figure S1: Bioinformatic analysis of the expression of two validated NRF2 transcriptional signatures in normal and tumour tissue., Figure S2: The mRNA levels for NQO1 and AKR1B10 in the different cell lines were quantified using real-time PCR, Figure S3: Scatter plots showing drug sensitivity score (DSS) values of DLD1 WT and GOF cells with cell viability (CellTiter Glo, CTG, upper panel) and of cell toxicity (CellTox Green, CTX, lower panel) readouts. and Figure S4: NRF2-WT and GOF DLD1 cells were exposed to increasing concentrations of AT9283 as indicated. Supplementary Table S1: Quality Control all plates, Table S2: Raw Data, Table S3: Cell viability complete screening, Table S4: Cell cytotoxicity complete screening.
