# Supplementary material for: High NRF2 Levels Correlate with Poor Prognosis in Colorectal Cancer Patients and with Sensitivity to the Kinase Inhibitor AT9283 In Vitro
Source: Biomolecules. 2020 Sep 25;10(10):1365. doi: 10.3390/biom10101365 (PMC7600603; doi:10.3390/biom10101365)
Supplement: Supplementary file 1 [file biomolecules-10-01365-s001.zip › biomolecules-933425 - supplementary/Supplementary figures.pdf]

S1A

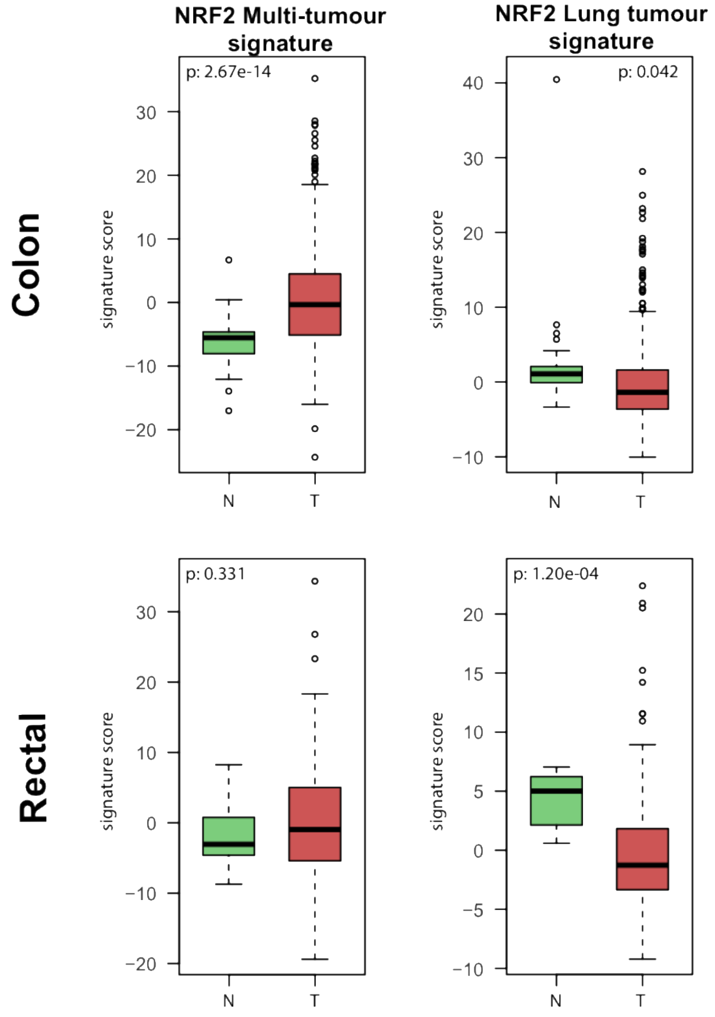

Suppl Fig 1

Figure 1. A. Bioinformatic analysis of the expression of two validated NRF2 transcriptional signatures in normal and tumour tissue. The expression of a multi-tumour NRF2 signature (left), or a lung cancer NRF2 signature (right) was assessed in colon (upper row) and rectal (lower row) cancers (T) and normal (N) tissues of the TCGA project. p: p-value of the Welch's t-test.

S1B

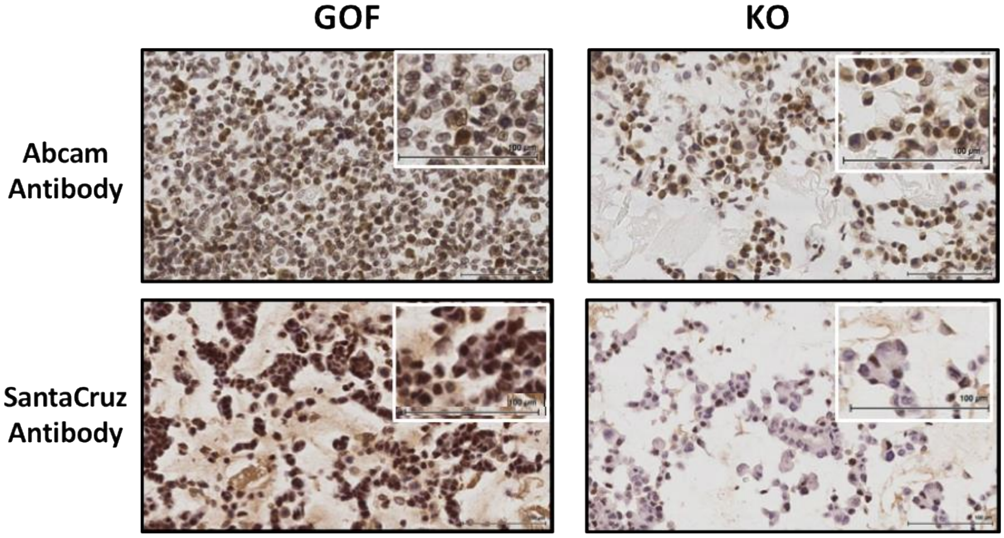

S1C

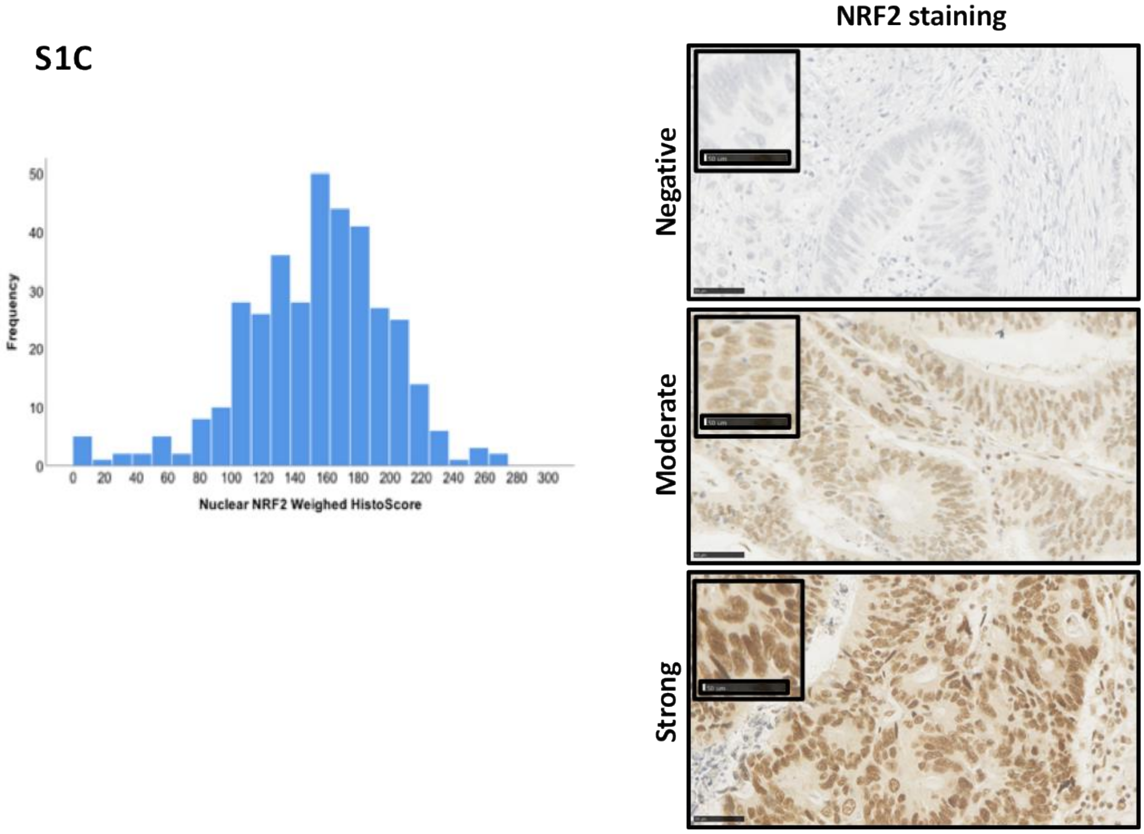

Suppl Fig 1

Figure 1. B. The top 2 panels show the expression pattern for NRF2 when compared in NRF2-GOF DLD1 cells versus NRF2-KO cells stained with the Abcam antibody. The bottom 2 panels show the expression pattern for NRF2 when compared in NRF2-GOF DLD1 cells versus NRF2-KO cells stained with the Santa Cruz antibody.

Figure 1. C. *Left panel*: Histogram displays range of expression observed for nuclear NRF2. *Right panels*: Representative examples showing nuclear NRF2 expression pattern classified as negative, moderate and strong staining.

S1D

		NRF Nuclear expression		
		Low (n=116)	High (n=69)	*P*
**Age**	<65	84	44	0.924
	>65	155	83	
**Sex**	Female	120	61	0.692
	Male	119	66	
**Tumour site**	Colon	181	89	0.245
	Rectum	58	38	
**T-stage**	1	7	8	0.166
	2	30	21	
	3	134	63	
	4	68	35	
**Differentiation**	Mod/well	207	114	0.376
	Poor	32	13	
**Venous invasion**	Absent	156	92	0.160
	Present	83	35	
**Peritoneal involvement**	No	171	89	0.768
	Yes	68	38	
**Proliferation Index**	Low	65	23	**0.038**
	High	173	102	
**MMR Status**	Competent	192	112	0.044
	Deficient	46	15	
**Tumour Necrosis**	Low	154	82	0.903
	High	85	44	
**Tumour budding**	Low	151	74	0.694
	High	70	31	
**Tumour Stroma Percentage**	Low	167	89	0.528
	High	63	34	
**Klintrup-Makinen grade**	Strong	163	88	0.464
	Weak	76	39	
**mGPS**	0	109	52	0.237
	1	44	21	
	2	33	8	

Suppl Fig 1

Figure 1. D. Chi square analysis demonstrating associations between low and high nuclear NRF2 expression and known clinicopathological characteristics.

S2A

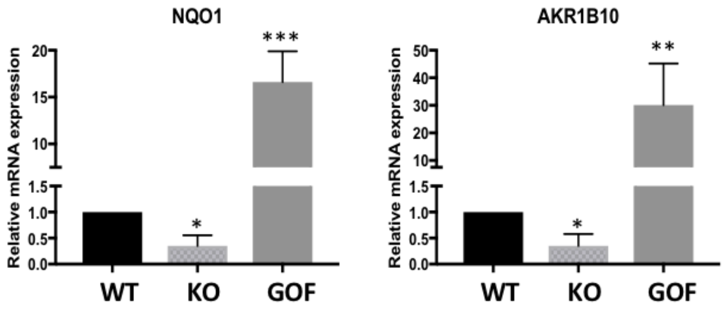

Suppl Fig 2

Figure 2. A. The mRNA levels for NQO1 and AKR1B10 in the different cell lines were quantified using real-time PCR. The data were normalized using β-actin as an internal control. Data represent means ± SD (n=3) and are expressed relative to the WT cells.

S3A

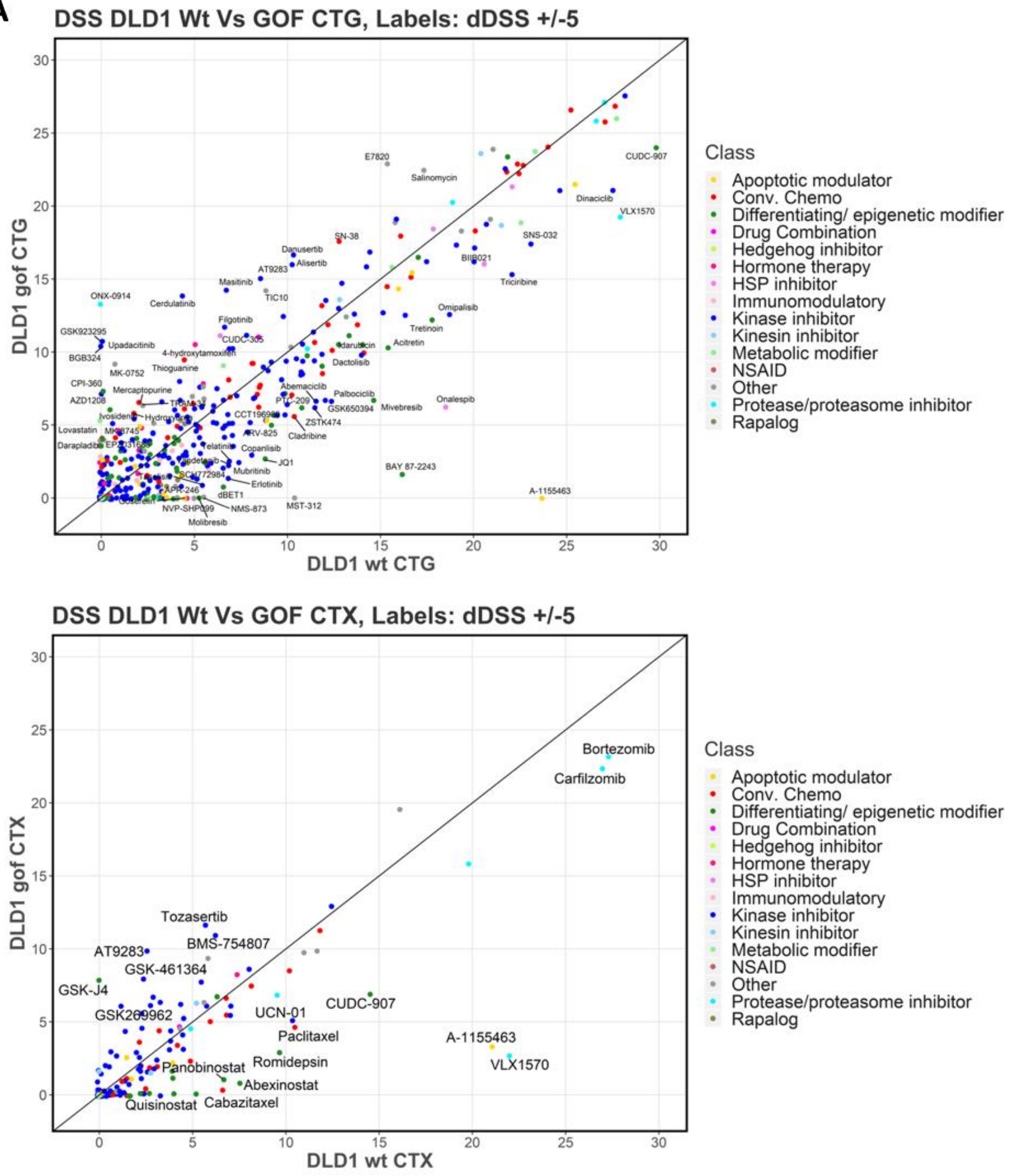

Suppl Fig 3

Figure 3. A. Scatter plots showing drug sensitivity score (DSS) values of DLD1 WT and GOF cells with cell viability (CellTiter Glo, CTG, upper panel) and of cell toxicity (CellTox Green, CTX, lower panel) readouts. Labels are shown only for compounds which have dDSS (DSS GOF-DSS wt) above or below 5.

S4A

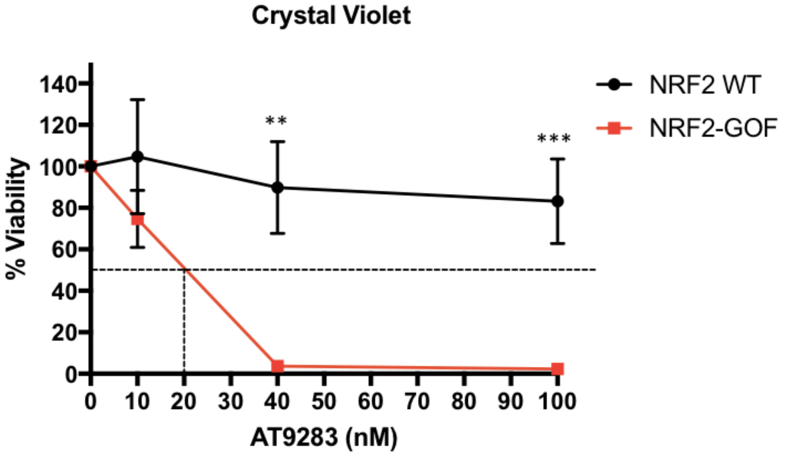

S4B

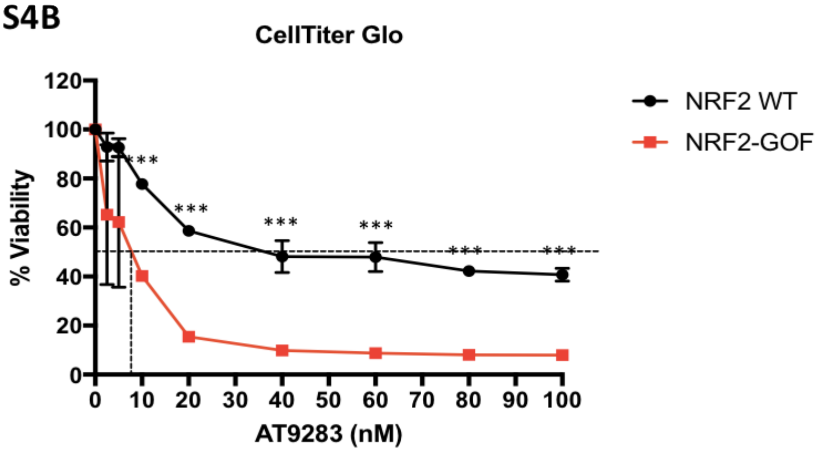

S4C

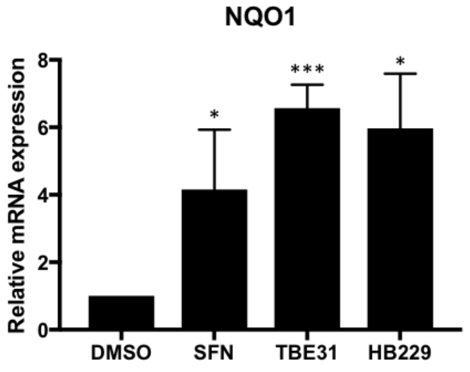

Suppl Fig 4

Figure 4. A. NRF2-WT and GOF DLD1 cells were exposed to increasing concentrations of AT9283 as indicated. After five days, cell viability was measured using Crystal Violet staining. Data represent means ± SD (n=7) and are expressed relative to the DMSO control which was set as 100%.

Figure 4. B. NRF2-WT and GOF DLD1 cells were exposed to increasing concentrations of AT9283 as indicated. After five days, cell viability was measured using CellTiter Glo. Data represent means ± SD (n=3) and are expressed relative to the DMSO control which was set as 100%.

Figure 4. C. DLD1 cells were treated with either DMSO, SFN 5 μM, TBE31 100 nM or HB229 10 μM. 16 hours later, the mRNA levels for NQO1 were quantified using real-time PCR. The data were normalized using β-actin as an internal control. Data represent means ± SD (n=3) and are expressed relative to the DMSO treated cells.
